# Seasonal Changes in Bird Species and Feeding Guilds along Elevational Gradients of the Central Himalayas, Nepal

**DOI:** 10.1371/journal.pone.0158362

**Published:** 2016-07-01

**Authors:** Hem Bahadur Katuwal, Khadga Basnet, Bhaiya Khanal, Shiva Devkota, Sanjeev Kumar Rai, Jyoti Prasad Gajurel, Christoph Scheidegger, Michael P. Nobis

**Affiliations:** 1 Central Department of Zoology, Tribhuvan University, Kathmandu, Nepal; 2 Small Mammals Conservation and Research Foundation, Kathmandu, Nepal; 3 Natural History Museum, Tribhuvan University, Kathmandu, Nepal; 4 Central Department of Botany, Tribhuvan University, Kathmandu, Nepal; 5 Swiss Federal Research Institute WSL, Birmensdorf, Switzerland; University of Colorado, UNITED STATES

## Abstract

The Himalayas are a global hotspot for bird diversity with a large number of threatened species, but little is known about seasonal changes in bird communities along elevational gradients in this region. We studied the seasonality of bird diversity in six valleys of the Central Himalayas, Nepal. Using 318 plots with a 50 m radius, located from 2200 to 3800 m a.s.l., and repeated sampling during different seasons (mainly pre-monsoon, monsoon, and post-monsoon), we analyzed 3642 occurrences of 178 species. Birds classified in the literature as resident were more species-rich than migratory birds (140 vs. 38 species). In all six valleys and within the studied elevation range, species richness of all birds showed a peak at mid-elevation levels of 2600 or 3000 m a.s.l. Similar patterns were found for the most species-rich feeding guilds of insectivores (96 species) and omnivores (24 species), whereas the species richness of herbivores (37 species including frugivores) increased towards higher elevations. Among these feeding guilds, only species richness of insectivores showed pronounced seasonal changes with higher species numbers during post-monsoon season. Similarly, individual bird species showed distinct spatio-temporal distribution patterns, with transitions from species dominated by elevational differences to those characterized by strong seasonal changes. In an era of climate change, the results demonstrate that individual bird species as well as feeding guilds might greatly differ in their responses to climate warming and changes in the seasonality of the precipitation regime, two aspects of climate change which should not be analyzed independently.

## Introduction

High mountain environments are characterized by considerable variation in geology, topography, climate, and land-cover along elevational gradients [1–3] and are known to feature a larger number of species than expected in a given area [4]. This characteristic also applies to birds [5] and is especially pronounced in the Himalayas, which are a global hotspot for bird species and contain a large number of threatened taxa [6–8].

Species richness and the composition of birds often change rapidly with elevation [9, 10], which makes these gradients well suited for studying the responses of bird communities to different environmental factors [2, 11]. For large elevational gradients, especially in humid climates [11], studies have often shown a humped-shaped relationship between bird species richness and elevation ([10, 12–16], but see [17–19]). Although climate, productivity, mass effects, species-area relationships, mid-domain effects, geomorphic constraints, evolutionary history, habitat structure, and human-induced disturbances have been underlined as important factors contributing to such mid-elevation peaks in species richness (e.g. [11, 19–22]), the actual causes and their interplay are still not well understood.

Seasonal changes in climate are an additional prominent characteristic of mountain ecosystems that can influence the temporal dynamics of bird species richness and composition [9, 23–25]. Birds in mountain environments are sensitive to seasonal variation in climate, due to resource bottlenecks for food and water availability and to temperature regulation requirements [25–27]. In Nepal, seasonal migration of birds is closely linked to changes between the dry and monsoon seasons. Summer migration usually starts between March and May (pre-monsoon season; sometimes migration is extended to monsoon season in June and July) and winter migration starts during the post-monsoon season in September [28–30]. Almost two thirds (62%) of the 878 bird species of Nepal have been classified primarily as residents, but only a small number of these birds are actually sedentary and most of them are elevational migrants over short distances [29]. Analyses of bird guilds, defined as functional groups of species that use resources in a similar way [31], in other parts of the world have demonstrated that seasonal migration is often most prevalent in insectivores [32] and coincides with fruit ripening for frugivorous species [26, 33, 34].

Although the Central Himalayas are recognized as a global hotspot of bird biodiversity, this region is still relatively unexplored in comparison to other mountain ranges (but see [8, 17, 21, 28, 29, 35–39]). This is especially true for species at higher elevations and regarding the seasonality of species occurrences. Species richness and composition of birds in the Central Himalayas can change rapidly with elevation and between the dry and wet season [21, 28, 29]. In an era of climate change, many studies have analyzed elevational patterns and the upwards shifts of species distributions in the context of climate warming [40–43] but the relevance of changes in precipitation is still largely ignored [44]. The main objective of our study, therefore, was to explore monsoon-driven seasonal changes in bird diversity along elevational gradients of the Central Himalayas in Nepal to address the following questions: (1) To what extent do individual bird species as well as functional species groups, i.e. migration and feeding guilds, show distinct elevational and seasonal distributions? (2) Which possible consequences of climate change can be inferred by using (a) distinct elevational distribution patterns as an indicator for temperature sensitivity and (b) distinct seasonality as an indicator for sensitivity to the monsoon-driven precipitation regime of the Central Himalayas?

## Materials and Methods

### Ethics Statement

Authorization to carry out the fieldwork in all the valleys was granted by the Department of National Parks and Wildlife Conservation, Kathmandu, and The National Trust for Nature Conservation, Lalitpur and this study does not involve any handling of endangered or protected species.

### Study Area

**Fig 1:**
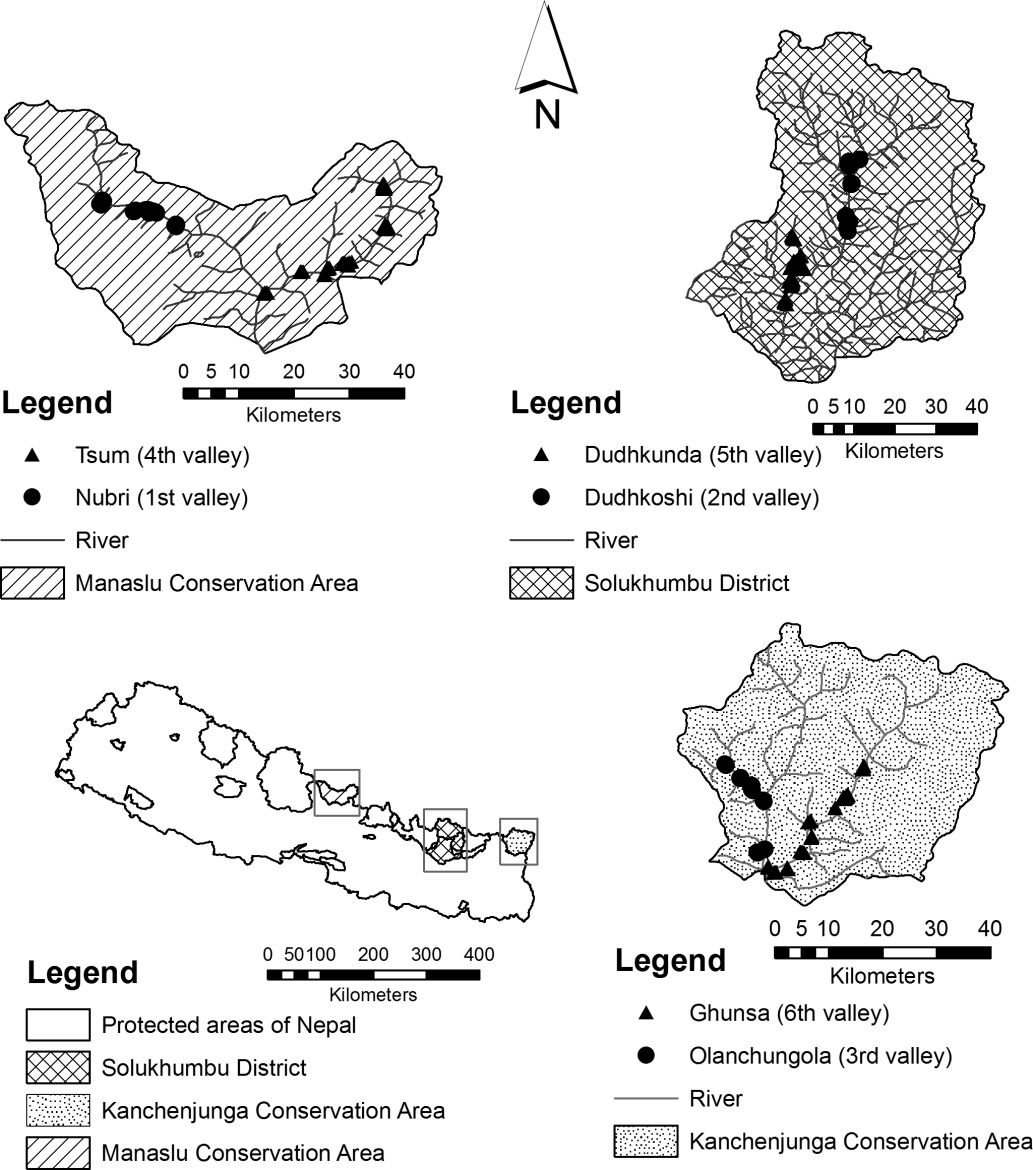
Overview of the three study regions. Map sections show the study sites in the investigated six valleys of the Central Himalayas, Nepal.

Seasonal dynamics of bird distributions along elevational gradients were studied in three regions of the Central Himalayas, Nepal (Fig 1). From west to east, the regions were: Manaslu Conservation Area (MCA) in the Gorkha district, the Dudhkoshi and Dudhkunda valleys (DDV) including Sagarmatha National Park (SNP) with its buffer zones in the Solukhumbu district, and Kanchenjunga Conservation Area (KCA) in the Taplejung district. In each of these three regions, two valleys were investigated: the Nubri (28.56927°N, 84.73417°E) and Tsum (28.48305°N, 85.04695°E) valleys in MCA, the Dudhkoshi (27.77505°N, 86.72220°E) and Dudhkunda (27.59188°N, 86.61712°E) valleys in DDV, and the Olanchungola (27.68068°N, 87.77882°E) and Ghunsa (27.59585°N, 87.87867°E) valleys in KCA. We assigned numbers to the valleys (one to six) in the following order: Nubri, Dudhkoshi, Olanchungola, Tsum, Dudhkunda and Ghunsa. BirdLife International has identified SNP and KCA as Important Bird Areas of Nepal, and this status has been proposed for MCA [38].

The bio-physiographic division of the Central Himalayas of Nepal is mainly comprised of three east-west running ranges: the Siwalik range (c. 1000–1500 m a.s.l.), the Mahabharat range or Lesser Himalayas (c. 1500–3000 m a.s.l.) and the Greater Himalayas (up to 8848 m a.s.l.) [45, 46]. The vegetation of the tropical/subtropical zone (up to c. 1000 m a.s.l.) is characterized by tree species like *Shorea robusta*, *Dalbergia sissoo* and *Bombax ceiba*, whereas *Schima wallichii*, *Castanopsis indica*, *Pinus roxburghii*, and *Alnus nepalensis* are common within the warm temperate zone (c. 1000–2000 m a.s.l). *Quercus* spp., *Juglans regia* and *Rhododendron* spp. are found within the cool temperate zone (c. 2000–3000 m a.s.l), *Abies spectabilis*, *Pinus wallichiana*, *Betula utilis* and *Rhododendron* spp. are characteristic of the subalpine zone (c. 3000–4100 m a.s.l), and finally *Juniperus* spp. and *Rhododendron* spp. dominate the alpine zone (>4100 m. a.s.l) including the tree line at *c*. 4000 m a.s.l. [47, 48]. The local human population is mostly Buddhist and is closely related to Tibetan communities, and cultural practices are similar in all of the regions studied. The Dudhkoshi valley of SNP, the Nubri valley of MCA and the Ghunsa valley of KCA have more human influence than other valleys, due to the establishment of major trekking routes. Slash and burn forest management practices were more common in KCA than in the other areas. Major rivers in the study area include Budigandaki and Siyar in MCA, Salleri and Dudhkoshi in DDV, and Tomor and Ghunsa in KCA [49].

### Sampling Design

In each valley, we investigated an elevational gradient of 1600 m, starting at 2200 m a.s.l. and ending at the highest human settlements, which were close to the forest line at 3800 m a.s.l. Although the Central Himalayas exhibit a much larger elevational gradient, according to Paudel and Šipoš [21], this range covers the highest bird species richness in Nepalese Himalaya. Sampling sites were established on both sides of the valleys at 2200, 2600, 3000, 3400, and 3800 m a.s.l. For each elevation level and valley side, four patches of different land use types were selected: natural forest, exploited forest, meadow and cultivated land [50]. We used these different major land use types to control for land use effects and to maximize the number of observed bird species by increasing the investigated habitat diversity. Natural forests were identified as forests with either pristine character or with a low anthropogenic influence, in most cases far-away from settlements; exploited forest showed an intensive exploitation for livestock grazing or for fuel and fodder collection; meadows showed less than 20% tree coverage and were frequently grazed by domestic livestock like yaks, goats, cows or horses; finally, cultivated lands, were intensively managed, terraced, fertilized, irrigated and yearly ploughed areas [50]. In each land use patch, circular plots were established for the observation of bird species. Thus, there were eight plots at each elevation level of a valley (within four land use types on each side of the valley), 40 plots per valley, and 120 plots in the first valley of the three regions (valleys 1 to 3). During a second round of field work, we extended our study design by adding a second valley in each region (valleys 4 to 6), each with two plots for each land use patch, resulting in 240 plots for these three new valleys and 360 plots in all six valleys. Due to bad weather conditions and missing land use types, we could only establish 314 of these plots. Field work was carried out between March 2011 and April 2013, with the first visits to valleys one to three in 2011 and to valleys four to six in 2012 and 2013. By April 2013, valleys one to three had been visited three times and valleys four to six had been visited twice during different seasons. Altogether, we were able to conduct 15 visits to the six valleys, with seven visits during pre-monsoon season, four visits during monsoon season, three visits during post-monsoon season, and one visit during winter (S1 Table).

On each land use patch, we established a circular plot with a radius of c. 50 m and spent 30 minutes on the plot to record the presence of bird species. Our subsequent analyses were based on species lists of the plots and, therefore, were not affected by multiple observations of the same individuals. In comparison to the fixed radius point count method [51–54], we used a larger plot area, a longer observation time, and an observer walking around on the plot to increase the number of observed species. Measuring tape was used to assess the distances from the central point in the field. Since birds within the elevation range of our study are active throughout the day, bird observation was done between 7:00 a.m. to 11:00 a.m. in valleys one to three and extended to 2:00 p.m. in valleys four to six due to the larger number of plots. All birds heard or seen within the radius were recorded, including flying ones in cases where they landed in the plot area. We used two field guides [28, 29], two binoculars (Nikon 10x50, Bushnell 10x42), and a digital camera (Canon-SX30IS Power shot with 35x zoom) for identification and documentation.

### Climate

We extracted climate data from WorldClim [55] for the coordinates of the plots to roughly describe climate conditions (mean annual precipitation and mean annual temperature) (S1 Fig). Both mean annual temperature and mean annual precipitation clearly decrease with elevation. In contrast to the similar temperatures in all three regions, annual precipitation was much higher in two regions (up to ≥ 2000 mm/year in DDV and KCA) in comparison to Nubri valley in MCA (maximum of about 1000 mm/year). The second valley of MCA (Tsum) had to be omitted from this climate characterization because of a mismatch between GPS-data and the WorldClim climate layers (S1 Fig).

### Data Analysis

Feeding guilds were classified into herbivores (frugivores feeding mostly on fruits, seeds, tubers, roots and grains), carnivores, omnivores, insectivores, and nectarivores, based on the diet descriptions available in Grimmett *et al*. [29, 30] and Basnet *et al*. [39]. The migration types were also based on Grimmett *et al*. [29, 30]. In contrast to numerous species classified as resident, different types of visitor and migratory birds showed very low species numbers in our study (S2 Fig). Therefore, we classified bird species as resident or migratory (defined as non-resident) to have a dataset that was more balanced for analyses.

Analyses were conducted in three steps, considering (1) elevational distribution patterns, (2) seasonal changes in bird diversity, and (3) the joint analysis of both elevational and seasonal effects on species diversity and single species distributions. For all analyses, we used presence–absence data of bird species at the plot level (i.e., one species list for a given plot and season) and two different datasets: all data (dataset A) and data from all three visits of the valleys Nubri (MCA) and Dudhkoshi (DDV) because these two valleys were consistently sampled during pre-monsoon, monsoon and post-monsoon seasons (dataset B). Following an initial comparison of species richness and of the proportion of species observations between migration types and feeding guilds, we calculated the average number of species in each of these groups at the plot level along the elevational gradient. For dataset B, we then calculated sample-based rarefaction curves [56] to compare species richness between different seasons for all birds and for individual feeding guilds. Finally, we analyzed elevational and seasonal effects together. Based on dataset B, we applied variation partitioning [57] with R-squared as a goodness-of-fit measure to detect independent effects of the season (pre-monsoon, monsoon and post-monsoon as categorical predictors) and elevation (levels 1 to 5 as linear and quadratic terms) to explain the variability in species observations for feeding guilds and for individual bird species. In addition, we used the same seasonal and elevational predictors in a multiple linear regression framework with AIC-based backward model selection to further evaluate elevational and seasonal effects on the number of observations for single species and for feeding guilds.

Rarefaction curves were calculated using EstimateS 9.1.0 [58]. All other analyses were carried out using R version 3.2.1 [59], with the R-package ‘raster’ for WorldClim analysis.

## Results

**Fig 2:**
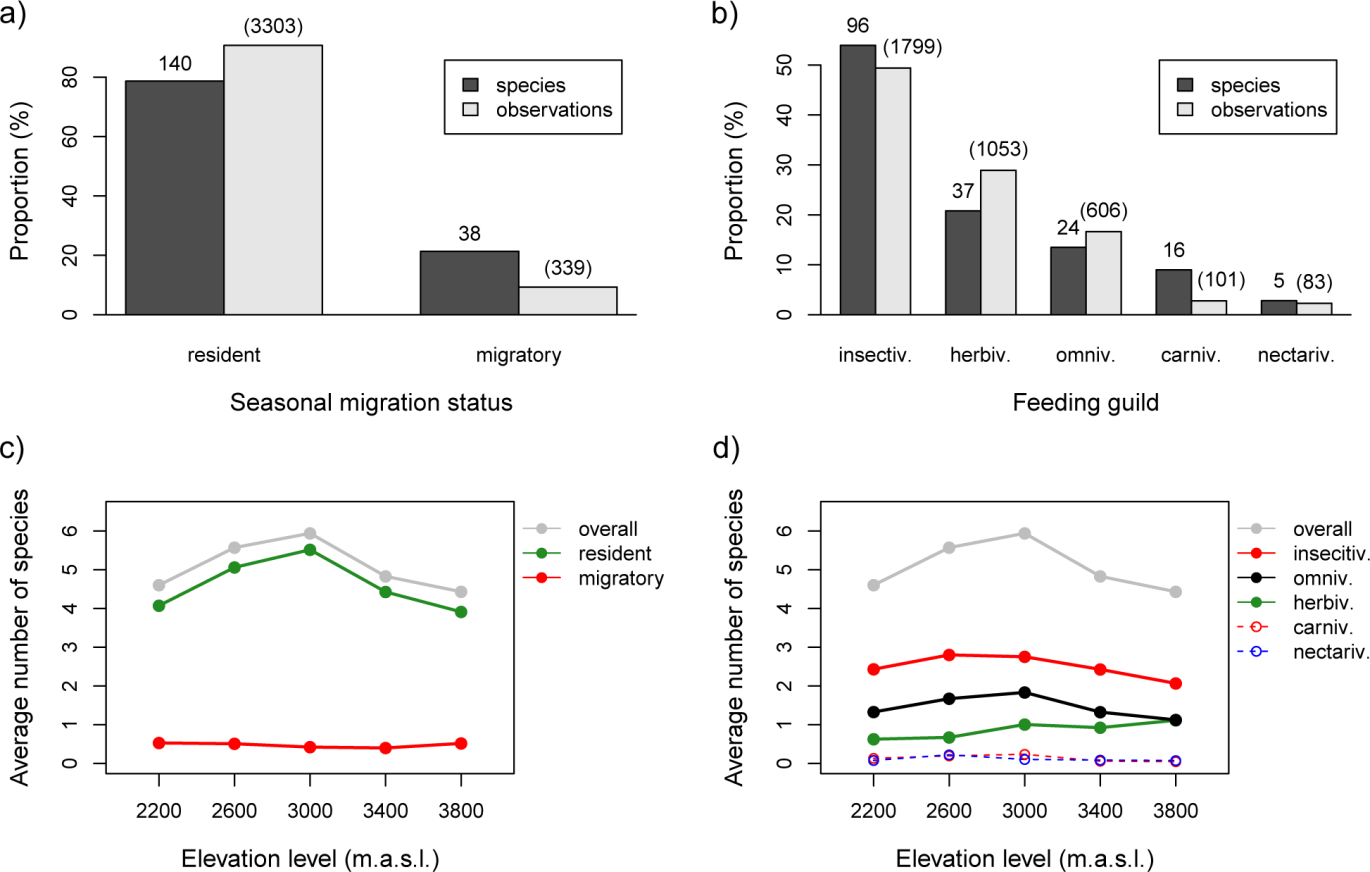
Overall species richness and number of species observations per plot for different species groups and along the elevational gradient. Species richness and sum of species observations per plot for (a) different migration types and (b) different feeding guilds, both based on the entire dataset of 314 plots. The average overall number of bird species per plot along the elevational gradient for different migration types (c) and feeding guilds (d) are shown.

In 314 plots, we recorded 3642 individuals of birds belonging to 178 species (see S2 Table). At the plot level, resident species had greater total species richness and a larger number of species observations than migratory birds (Fig 2A and S2 Table). With respect to feeding guilds, we recorded the largest number of insectivorous species, followed by herbivores and then omnivores (Fig 2B).

### Species Richness along Elevational Gradients

Summarized over all valleys, the average number of bird species per plot and the number of species classified as residents both showed a peak at the 3000 m a.s.l. elevation level, while the average number of migratory species was consistently low across the studied elevational gradient (Fig 2C). For single valleys, the maximum average number of species per plot was found at the elevation level 3000 (3 valleys) or 2600 m a.s.l. (3 valleys). In each valley, the average species number in plots at 2600 and at 3000 m a.s.l. was larger compared to the other elevation levels (*P* < 0.05 in all one-sided t-tests). Using data from all valleys, the average number of species per plot at 2600 and 3000 m a.s.l. was significantly larger than the average number of species at lower as well as higher elevation levels (*P* < 0.0001 for both one-sided t-tests). Among the three most frequent feeding guilds, insectivores and omnivores showed the greatest average species richness per plot, again occurring at 2600 and 3000 m a.s.l., whereas the average number of herbivore species showed an overall increase with elevation (Fig 2D). Very few species of carnivores and nectarivores were recorded.

### Seasonal Changes in Species Richness

**Fig 3:**
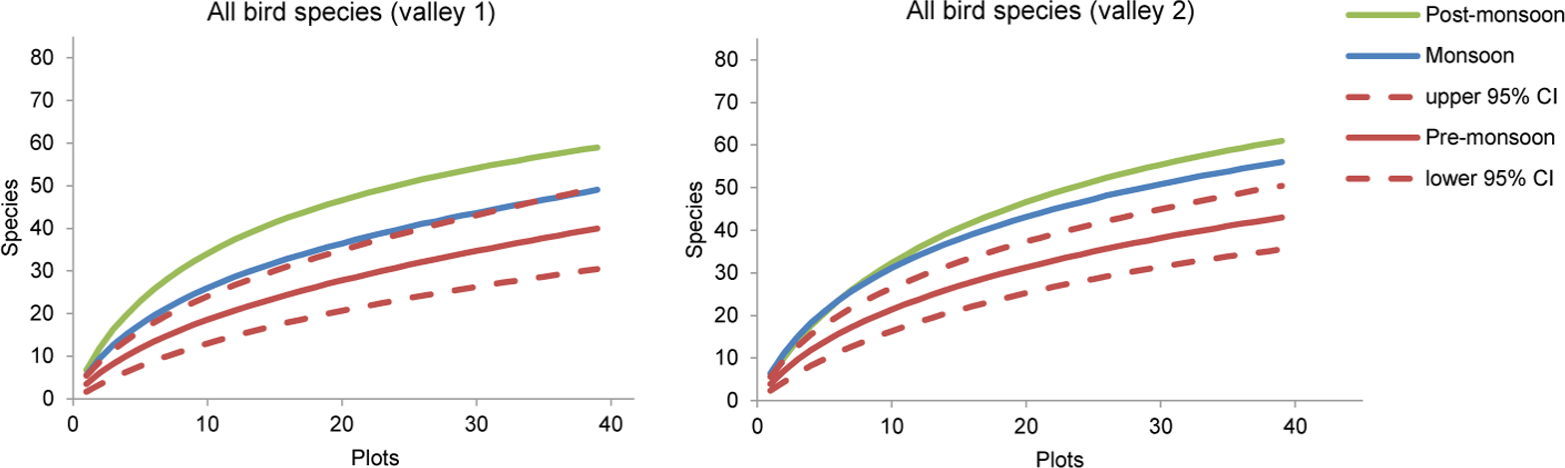
Sample-based rarefaction curves of estimated species richness of birds in the first two valleys Nubri (MCA) and Dudhkoshi (DDV) during pre-monsoon, monsoon, and post-monsoon seasons. Dashed lines indicate the 95% confidence interval (CI) of pre-monsoon estimates.

During all seasons, species richness of birds increased with increasing sample size without a clear saturation (Fig 3 and S3 Fig). For valleys 1 and 2 of dataset B, rarefaction curves indicated that the lowest richness of all bird species occurred during pre-monsoon season, with increasing species richness during monsoon and post-monsoon seasons (Fig 3). These seasonal changes were confirmed by data from the other valleys (S3 Fig), with the exception of the third visit to valley 3, which showed higher richness values during pre-monsoon than monsoon seasons, although these values still fall within the 95% confidence interval of the monsoon curve (not shown). In valley 6, species richness during winter was even lower than during pre-monsoon season (S3 Fig).

**Fig 4:**
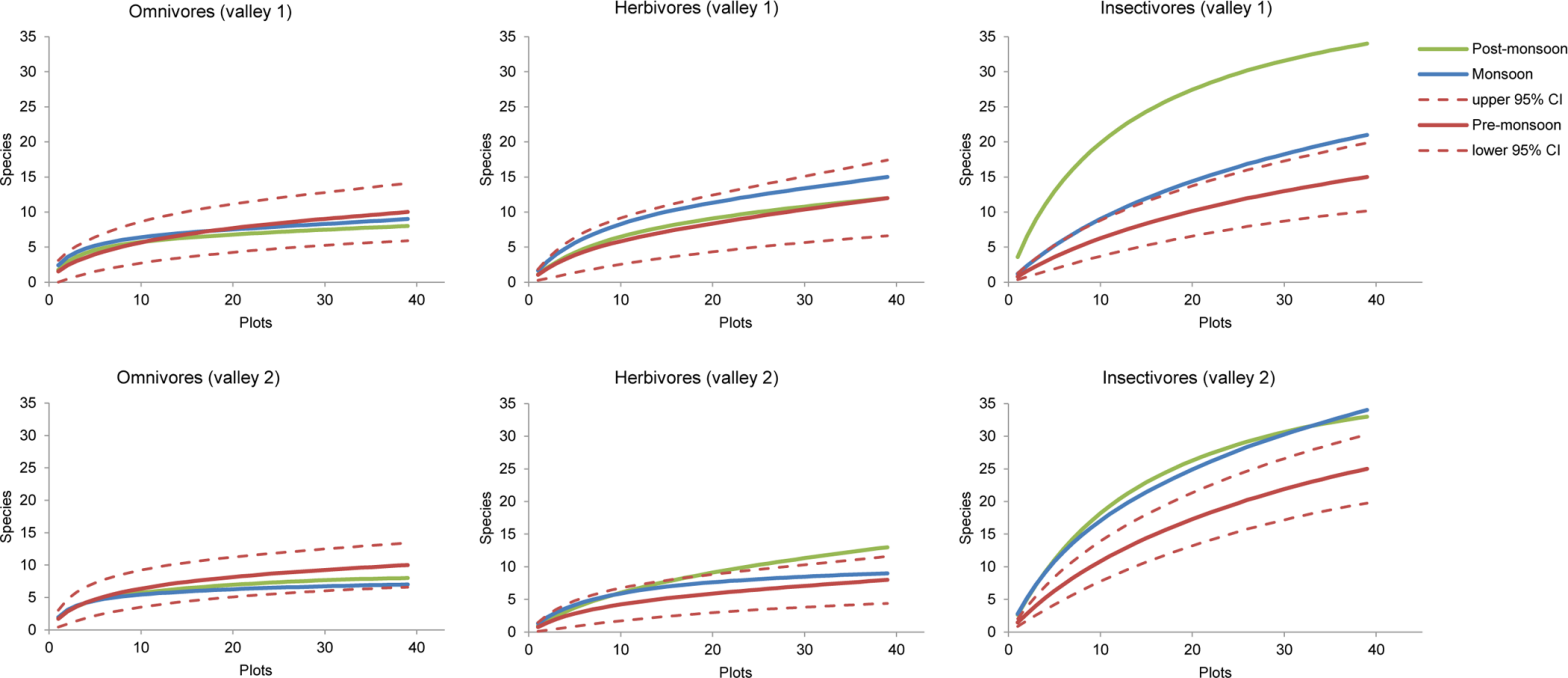
Sample-based rarefaction curves of estimated species richness of birds in the first two valleys Nubri (MCA) and Dudhkoshi (DDV) for the three most frequent feeding guilds (i.e., insectivores, herbivores, and omnivores) and for the pre-monsoon, monsoon, and post-monsoon seasons. Dashed lines indicate the 95% confidence interval (CI) of pre-monsoon estimates.

Corresponding results for the three most frequent feeding guilds revealed only minor seasonal changes in species richness of omnivorous and herbivorous birds, whereas the number of insectivorous bird species strongly increased from pre-monsoon to post-monsoon season (Fig 4).

### Elevational Patterns vs. Seasonal Changes

**Fig 5:**
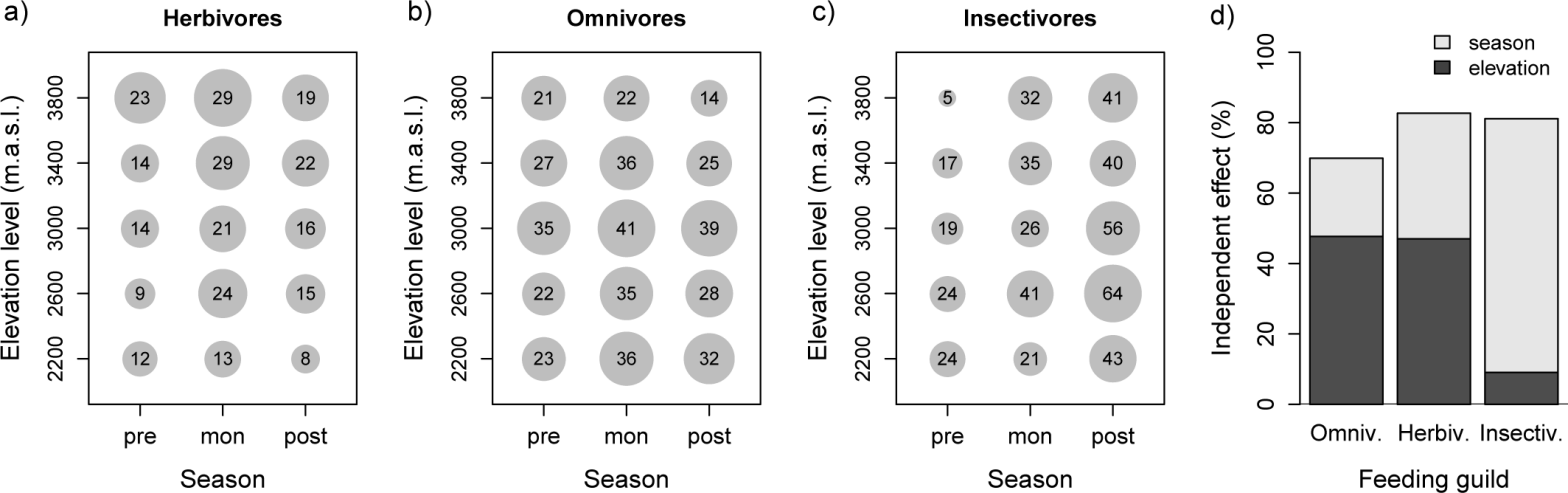
**Seasonal distribution of the three most frequent feeding guilds, i.e., (a) herbivores, (b) omnivores, and (c) insectivores along the elevational gradient expressed as cumulative number of species observations per plot.** The diameter of the circles illustrates the relative number of species observations. Variation partitioning and the independent effects of elevation and season in explaining these distributions are given in (d).

Seasonal changes in the cumulative number of species observations per elevation level are shown in Fig 5, which includes the three most frequent feeding guilds and is based on dataset B. For the investigated elevation range, species observations of herbivores increased with elevation and were more frequent during the monsoon season (Fig 5A). In contrast, the species observations of omnivores were quite constant among the seasons (Fig 5B). In addition, insectivores showed a pronounced seasonal change, with larger numbers of species observations during the post-monsoon season, especially at higher elevation levels (Fig 5C). The independent effects of both elevational and seasonal effects are summarized in the variation partitioning results presented in Fig 5D. The independent effect of the studied elevational range was stronger than that of the season for omnivores and herbivores, whereas the distribution pattern of insectivores was mainly driven by the season. However, when tested with an AIC-based stepwise regression of the full model, the effects of both elevation and season were included in the final model for omnivores (*P* < 0.05 for both effects; adjusted *R²* = 0.60) and herbivores (*P* < 0.001 for both effects; adjusted *R²* = 0.78), whereas only the seasonal effect was included for insectivores (*P* < 0.001; adjusted *R*² = 0.73).

**Fig 6:**
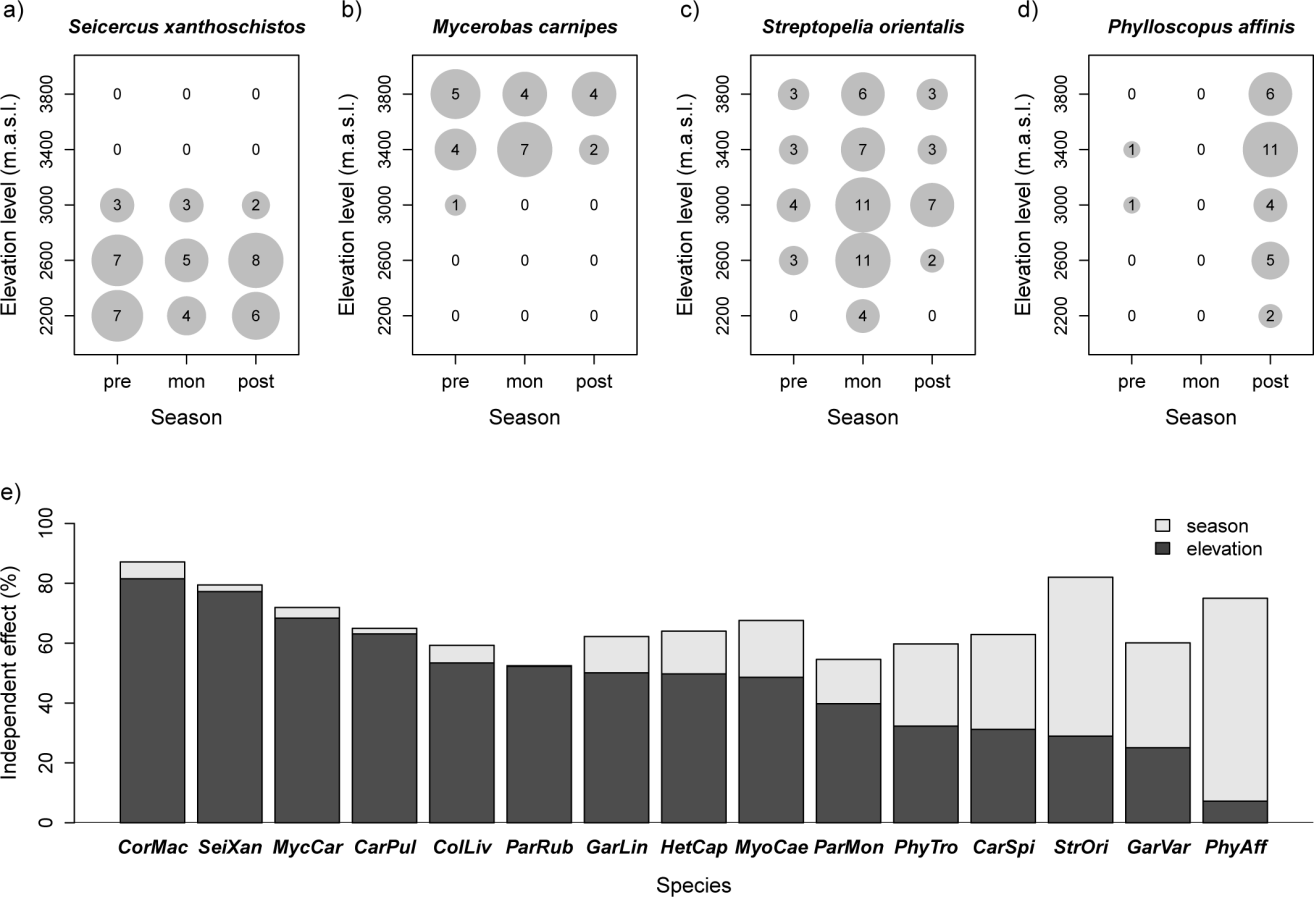
Contrasting seasonal distributions of individual bird species along the elevational gradient. (a-d) Distributions of four frequent bird species at different elevation levels and during different seasons. Numbers and the diameter of the circles illustrate the number of plots with the species observations. (e) Variation partitioning and the independent effects of elevation and season in explaining these distributions for the 15 most frequent bird species. For species abbreviations and additional distribution plots, see S2 Table and S4 Fig.

The corresponding analysis of the 15 most frequent bird species revealed contrasting spatio-temporal patterns for individual species (Fig 6A–6D, S4 Fig). For example, the cumulative number of species observations per plot for Grey-hooded Warbler (*Seicercus xanthoschistos)* and White-winged Grosbeak (*Mycerobas carnipes*) showed no major seasonal changes but pronounced elevational patterns, with occurrences only at the lower or upper end of the studied elevation range (Fig 6A–6B). In contrast, other birds like Oriental Turtle Dove (*Streptopelia orientalis)* and especially Tickell’s Leaf Warbler (*Phylloscopus affinis*) revealed strong seasonal changes, with the largest numbers of observations occurring in specific seasons (Fig 6C–6D). Again, independent effects calculated by variation partitioning were used to summarize the varying importance of elevation and season in explaining these spatio-temporal patterns of species occurrences (Fig 6E). Stepwise regression conducted on the full models showed that the main effect (elevation level or season) was included in the final model for all species (*P* < 0.05), and for five out of the 15 species both elevation and season were included as predictors in the final model (*P* < 0.05). These stepwise models explained between 39% and 82% of variation in species observations (58% on average).

## Discussion

Our study revealed pronounced differences in elevational and seasonal distributions of birds in the Central Himalayas of Nepal that varied with migration type, feeding guild and species identity.

### Species Richness along Elevational Gradients

Mid-elevation peaks in bird species richness have often been observed in mountain ranges worldwide [10, 12–14, 20, 60]. For the Central Himalayas of Nepal in particular, the peaks we observed in all valleys at the elevation levels of 3000 or 2600 m a.s.l. are consistent with Paudel and Šipoš [21], who reported the highest bird species richness at c. 2800 m a.s.l. However, the investigated elevation range and method clearly differed between the two studies. While we recorded bird species directly at the plot level using a standardized size and sampling effort, Paudel and Šipoš [21] used elevation range information from literature and interpolated species presence within 100 m elevational bands. Therefore, our study based on much smaller study sites and standardized sampling is complementary to that of Paudel and Šipoš [21] and confirms their results. In contrast, in another study where bird species richness of Nepal was also compiled from literature for even wider elevation bands of 500 m [17], richness showed an overall decrease towards higher elevations. Interpolated species richness values for elevation bands represent species pools and therefore the maximum number of potentially co-occurring species rather than representing local bird communities (see also [61]). Accordingly, if richness data are standardized for area or sampling effort, continuous declines in non-standardized species richness towards higher elevation can become hump-shaped, showing a mid-elevation peak [12]. It has also been argued that interpolations from literature are biased due to different sampling effort [21], which is generally higher at lower elevation. This bias might increase species richness at lower elevations and lead to a greater uncertainty of range limits at higher elevations. The fact that we observed some species more than 300 m above their upper elevation limits as described in Grimmett *et al*. [29] could be due to such uncertainties. However, recent upward shifts of species ranges in response to climate change (e.g. [40, 41]) have most likely occurred in the Himalayas, one of the world’s most vulnerable regions to climate change [62], which could also explain these observations. Occurrences clearly above previously reported species’ upper elevation limits by at least 300 m were made both for resident species, like White-browed Fulvetta (*Alcippe vinipectus*), Rufous Sibia (*Heterphasia capistrata*), Golden-spectacled Warbler (*Seicercus burkii*), Back-shouldered Kite (*Elanus caeruleus*), and Spotted Nutcraker (*Nucifraga caryocatactes*), and for migratory species, like Large-billed Leaf Warbler (*Phylloscopus magnirostris)* and Blue-capped Rock Thrush (*Monticola cinclorhynchus*).

Regarding feeding guilds, our results confirm previous studies (e.g. [3, 63]) showing the importance of food resources for the differentiation of bird species richness and composition along elevational gradients. Insectivorous birds often constitute the most species-rich feeding guild (e.g. [26, 64, 65]), and a mid-elevation peak in insectivore species richness has been found to coincide with food availability (e.g. [63]). Like in other mountain ranges (e.g. [3]), the species richness of herbivores declines at higher elevations in Nepal [29, 39], but such a decline was not detectable in our study due to the constraints of the investigated elevation range. Such differences in elevational richness patterns among feeding guilds also reflect evolutionary history, as feeding traits are assumed to have evolved early during species diversification, whereas species differentiation along elevational gradients was a more recent process [63].

Paudel and Šipoš [21] argued that factors related to human management are important drivers of the elevational gradient of bird species richness in Nepal. Our analyses focused on elevational vs. seasonal diversity patterns and did not include more direct drivers of bird diversity like climate or land cover. Nonetheless, we partly controlled for human management by sampling the same land use types at each elevational level. Together with the fact that human settlements were consistently present along the investigated elevational gradients, we are confident that human impact was not the major factor of the species richness patterns observed in our study.

### Seasonal Changes in Species Richness

Species richness significantly increased from pre-monsoon to the monsoon and post-monsoon seasons. This demonstrates that the precipitation regime driven by monsoon has a strong impact on the seasonal distribution and species richness of birds in this part of the Central Himalayas. However, there are clear differences between feeding guilds. Omnivorous birds with the lowest degree of diet specialization are apparently not as strongly affected by precipitation regime, and their rarefaction curves of species richness did not reveal any clear seasonality. Species richness of herbivores also showed only very weak seasonality in both valleys, although the mean annual precipitation in Nubri valley (MCA) is much lower than in Dudhkoshi valley and reaches less than 500 mm/year at lower elevation levels (S1 Fig). In contrast, insectivores clearly showed the strongest seasonal response, with the highest species numbers occurring during post-monsoon. As the most species-rich feeding guild, insectivores also caused the seasonal pattern in species richness of all birds. The seasonal changes in bird species richness can therefore be interpreted mainly as a response to a resource bottleneck for food during the pre-monsoon season (and winter) followed by improved food availability during the monsoon and post-monsoon seasons.

In contrast to the clear differences in mean annual precipitation of Nubri valley and Dudhkoshi valley (S1 Fig), each of the three main feeding guilds showed a similar seasonality in species richness for both valleys (Fig 4). This result indicates that the observed patterns are robust and valid for a wide range of mean annual precipitation. The higher species richness of insectivores in Dudhkoshi valley during pre-monsoon season, however, might be due to the higher mean annual precipitation in comparison to Nubri valley.

Finally, the fact that the sample-based rarefaction curves of seasonal species richness show no clear saturation indicates the presence of many rare bird species. This finding additionally confirms the reliability of our sampling design, which included a larger number of plots and land use types than other similar studies, in that the redundancy of species observations at the plot level was low.

### Elevational vs. Seasonal Changes

The comparison of elevational and seasonal effects on species richness, as well as single species distributions, demonstrated that both elevation and season are important for the characterization of bird diversity in the studied mountain valleys. Although our study covered only a small part of the whole elevational gradient of the Central Himalayas of Nepal, some major patterns of species distributions and feeding guilds were clearly link to elevation, while others were dominated by seasonality. In comparison to feeding guilds, the distribution patterns of individual species revealed an even higher variability. These distributions showed gradual but also surprisingly abrupt changes between seasons and elevation levels (Fig 6, S4 Fig). These abrupt changes were not caused by special conditions in the two analyzed valleys of Nubri and Dudhkoshi (dataset B). In the cases of Grey-hooded Warbler (*Seicercus xanthoschistos*; Fig 6A) and White-winged Grosbeak (*Mycerobas carnipes*; Fig 6B), the same strong elevational pattern occurred in all four remaining valleys (no occurrences of *Seicercus xanthoschistos* at the elevation levels above 3000 m a.s.l; no occurrences of *Mycerobas carnipes* at elevation levels below 3000 m a.s.l). Similarly, the increase of Tickell's Leaf Warbler (*Phylloscopus affinis*) presence during the post-monsoon season (Fig 6D) was confirmed by occurrences in the valleys of Tsum and Dudhkunda (17 species observations during the post-monsoon season but only three observations during the monsoon season and none during the pre-monsoon season). We therefore interpret these sharp seasonal and elevational changes as robust and ecologically meaningful results.

Elevation and season are of course only proxies for more direct environmental factors driving bird diversity. Because wider elevational gradients always reflect climate gradients, spatial patterns and seasonality of bird diversity might indicate how functional groups or individual species might differ in their response to climate change. Our data therefore contribute to a research field of conservation relevance that has attracted a large amount of interest in recent years (e.g. [66, 67]). With clear elevational patterns but no or weak seasonal distribution patterns (e.g. *Seicercus xanthoschistos* and *Mycerobas carnipes* in Fig 6A–6B), species probably profit from or compensate for climate warming by undergoing upwards shifts, assuming that the current distribution pattern is directly or indirectly driven by temperature. Although annual precipitation can vary significantly along elevational gradients, upwards shifts in species distributions have most often been related to climate warming [40–43]. One reason for this might be that contemporary changes as well as future projections of annual precipitation are less certain than those of temperature [68, 69]. In addition, current annual precipitation has been found to either increase or decline with increasing elevation. Similar changes in precipitation might therefore result in opposite shifts in bird diversity for different mountain ranges. For the Sierra Nevada Mountains, USA, for instance, downslope range shifts of birds have been reported and have been linked to increases in precipitation over the past century [44]. In contrast, within the elevation range of our study regions, increases in annual precipitation due to climate change might cause upwards shifts of species because precipitation generally decreases with elevation. In addition to these elevational patterns of bird distributions, our study showed clear seasonal and no or weak elevational distribution patterns for other bird species. Within the given elevation range, species like *Phylloscopus affinis* (Fig 6D) and insectivores as a functional group are probably more sensitive to seasonal changes in precipitation than to annual totals or means. In contrast to temperature, which is a key driver of seasonality in bird species in many temperate mountain ranges, seasonality in precipitation due to the monsoon season should be treated as a key climate factor influencing bird diversity in our study regions.

It is important to discuss some potential limitations of our study. First, our results are restricted to an elevation range between 2200 and 3800 m a.s.l., and we did not cover the entire elevation ranges or geographic distributions of the species. In particular, the partitioning between elevational and seasonal effects on species distributions has to be interpreted in the context of this spatial restriction. Second, due to logistical challenges in remote areas of Nepal and to the fact that the study was part of a larger multi-taxa project, sampling effort in the different seasons was not well balanced and very few sampling events were conducted in the winter season. This imbalance clearly influenced our analyses. For the spatio-temporal analyses of dataset B, however, we restricted the data to equally sample in pre-monsoon, monsoon, and post-monsoon seasons. Finally, we used coarse migration types and feeding guilds, due to missing information or otherwise too small numbers of species or observations for analyses. For a few species, the classification of migration type and feeding guild might be questioned and sometimes contradicted our empirical findings. At the level of our species groups, however, these uncertainties regarding single species probably did not have a major effect on the overall results of our study.

In summary, our study revealed pronounced elevational and seasonal differences in the distributions of both species richness and individual bird species in six valleys of the Central Himalayas, Nepal, that varied among migration types and feeding guilds. These patterns can be linked to contemporary climate gradients and climate seasonality. Since the migration behavior of short distance and elevational migrants is clearly less studied than that of long distance migrants (see [66,70, 71]), analyses of seasonal distributions along elevational gradients are also important for filling knowledge gaps regarding bird migration. They contribute to our understanding of how bird species might differ in their response to climate warming and to seasonal changes in the precipitation regime, two major aspects of ongoing climate change that should not be analyzed independently.

## Supporting Information

S1 Fig Mean annual temperature and precipitation of the three study regions of the Central Himalayas, Nepal. Mean annual temperature (a) and mean annual precipitation (b) based on WorldClim data from the locations of the point count stations. One valley (Tsum) had to be omitted because a clear elevational gradient was not detectable and the extracted elevations from WorldClim did not match the field measurements (mean absolute error MAE was 805 m, whereas MAE was 105 m for all other valleys).
(TIF)

S2 Fig Species richness of birds with different seasonal migration types, based on descriptions in Grimmett *et al*. (2000) for the 178 observed bird species. (TIF)

S3 Fig Sample-based rarefaction curves of estimated species richness of birds for the third to sixth valley during pre-monsoon, monsoon, and post-monsoon. Dashed lines indicate the 95% confidence interval (CI) of pre-monsoon estimates.
(TIF)

S4 Fig Seasonal distribution of individual bird species along the elevational gradients. The list of bird species with more than 25 presences that are not shown in Fig 6. Numbers indicate how many observations were made at the count station level and are also illustrated by the diameter of the circles; seasons are pre-monsoon (pre), monsoon (mon), and post-monsoon (post).
(TIF)

S1 Table Date and season of the visits to the six valleys in the Central Himalayas, Nepal. (DOCX)

S2 Table List of overall 178 species recorded and list of unexpected observations in six valleys of the Central Himalayas, Nepal. The feeding guild and migration classification, based on descriptions available in Grimmett *et al*. (2000, 2011) and Basnet *et al*. (2016). Valleys 1 to 6 represent Nubri, Dudhkoshi, Olanchungola, Tsum, Dudhkunda, and Ghunsa, respectively. Feeding guilds are carnivores (C), herbivores (H), insectivores (I), nectarivores (N), and omnivores (O). Unexpected observations occurred for: Blue-capped Rock Thrush (*Monticola cinclorhynchus*), Large-billed Leaf Warbler (*Phylloscopus magnirostris)*, White-browed Fulvetta (*Alcippe vinipectus*), Rufous Sibia (*Heterphasia capistrata*), Golden-spectacled Warbler (*Seicercus burkii*), and Spotted Nutcraker (*Nucifraga caryocatactes*), all of which were recorded at least 300 m above their upper elevation limits as described in Grimmett *et al*. (2000). The common lowland species Back-shouldered Kite (*Elanus caeruleus*) was recorded in DDV at 3000 m a.s.l., although Grimmett *et al*. (2000) indicate that the species is located up to 1550 m a.s.l. as a summer visitor in the Kathmandu valley.
(DOC)
