# Supplementary figures and images for: Seasonal Changes in Bird Species and Feeding Guilds along Elevational Gradients of the Central Himalayas, Nepal
Source: PLoS One. 2016 Jul 1;11(7):e0158362. doi: 10.1371/journal.pone.0158362 (PMC4930183; doi:10.1371/journal.pone.0158362)

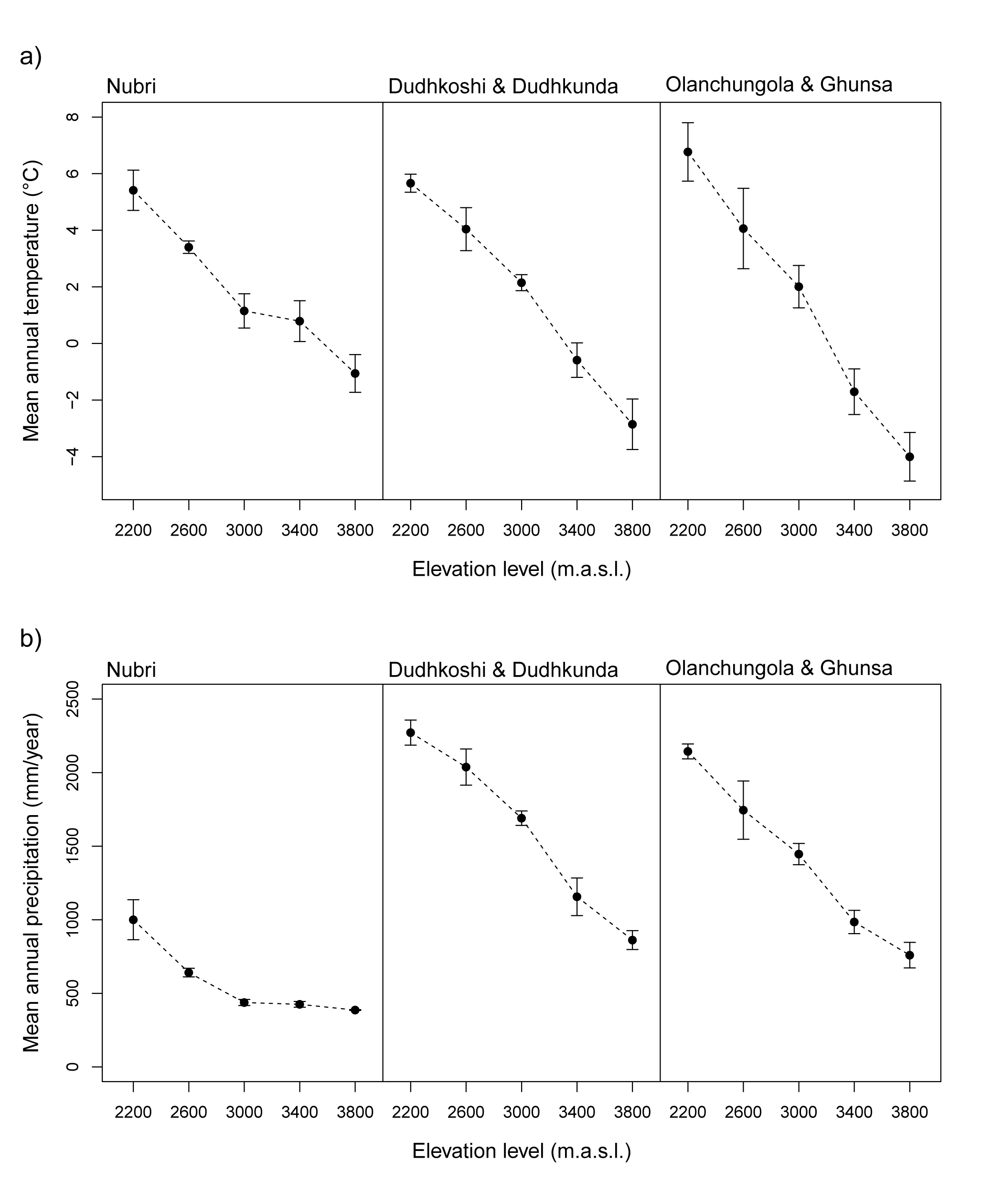

Supplement: S1 Fig — Mean annual temperature (a) and mean annual precipitation (b) based on WorldClim data from the locations of the point count stations. One valley (Tsum) had to be omitted because a clear elevational gradient was not detectable and the extracted elevations from WorldClim did not match the field measurements (mean absolute error MAE was 805 m, whereas MAE was 105 m for all other valleys). (TIF) [file pone.0158362.s001.tif]

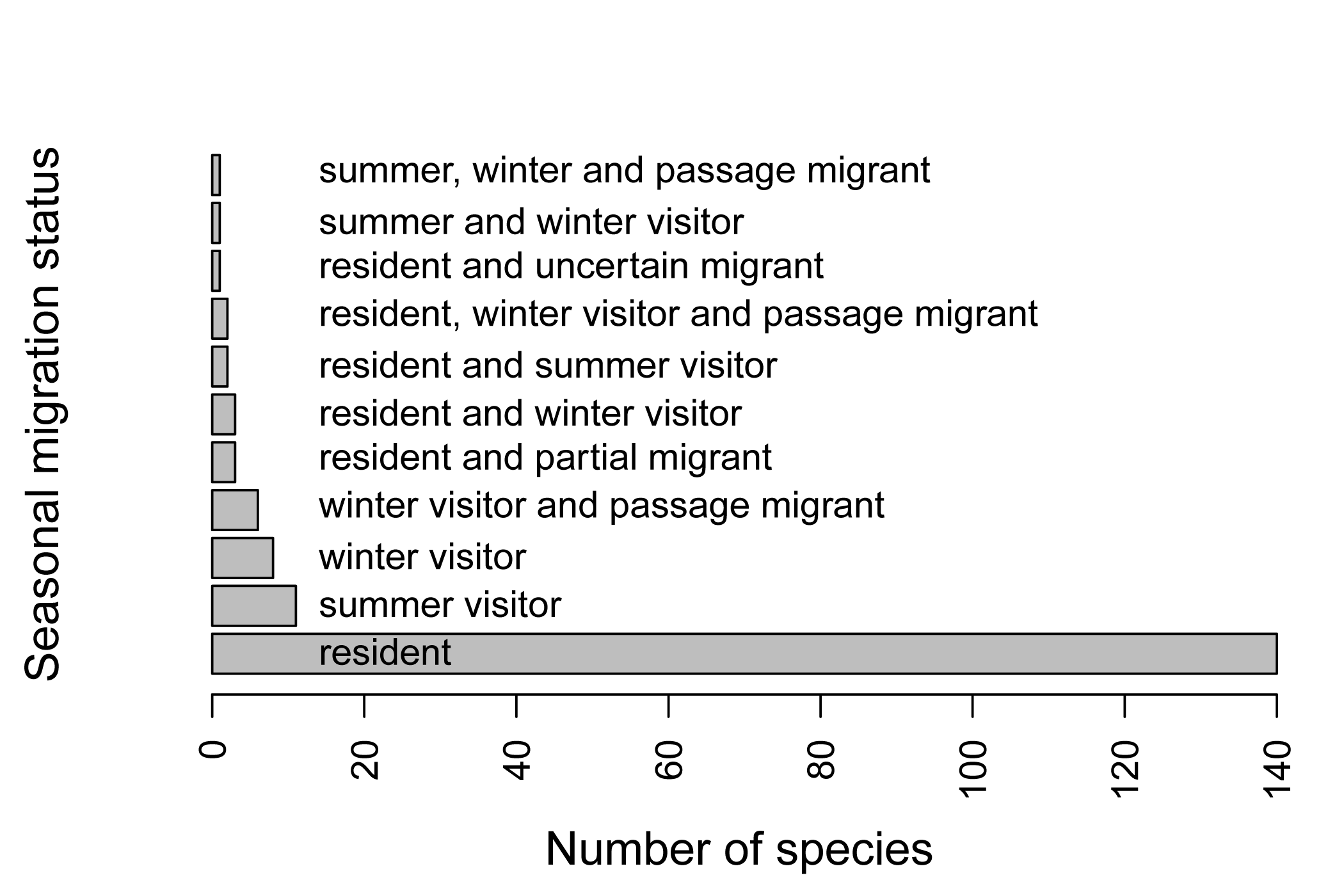

Supplement: S2 Fig — (TIF) [file pone.0158362.s002.tif]

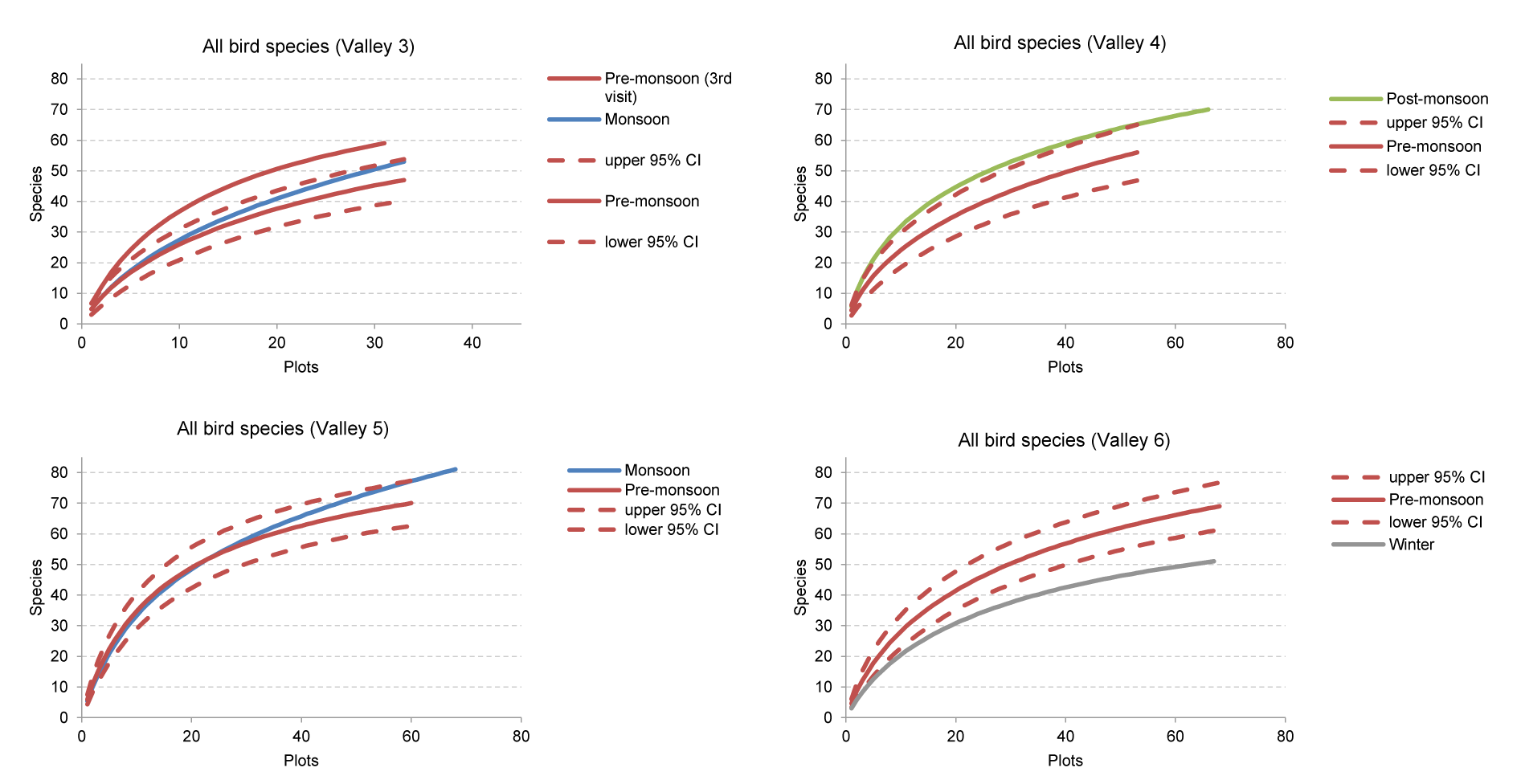

Supplement: S3 Fig — Dashed lines indicate the 95% confidence interval (CI) of pre-monsoon estimates. (TIF) [file pone.0158362.s003.tif]

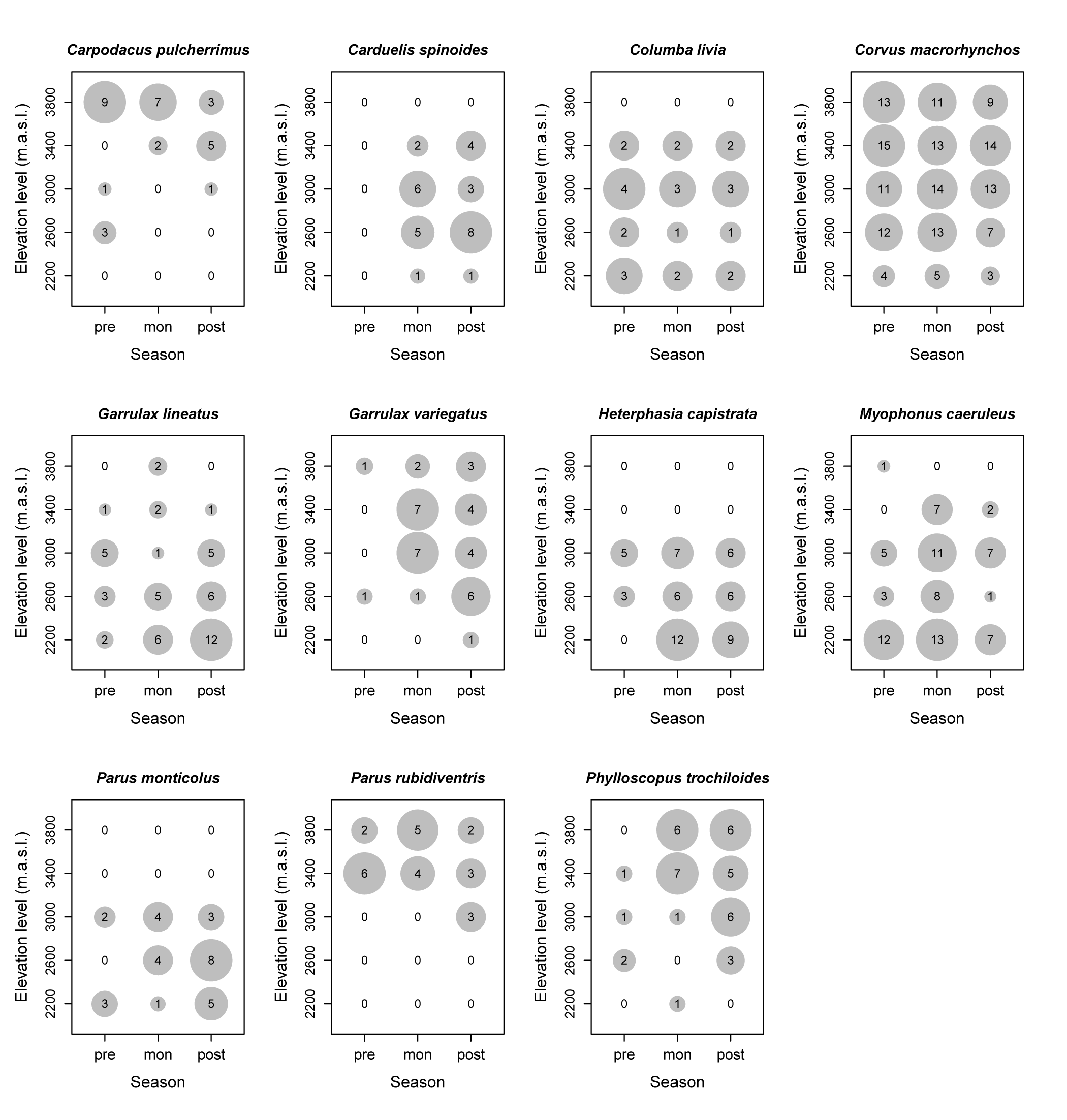

Supplement: S4 Fig — The list of bird species with more than 25 presences that are not shown in Fig 6. Numbers indicate how many observations were made at the count station level and are also illustrated by the diameter of the circles; seasons are pre-monsoon (pre), monsoon (mon), and post-monsoon (post). (TIF) [file pone.0158362.s004.tif]
