# Supplementary material for: Seasonal Changes in Bird Species and Feeding Guilds along Elevational Gradients of the Central Himalayas, Nepal
Source: PLoS One. 2016 Jul 1;11(7):e0158362. doi: 10.1371/journal.pone.0158362 (PMC4930183; doi:10.1371/journal.pone.0158362)
Supplement: S1 Table — (DOCX) [file pone.0158362.s005.docx]

**Supporting Information**

**S1Table.** **Date and season of the visits to the six valleys in the Central Himalayas, Nepal.**

| **Year** | **Month** | **Valley No.** | **Visit** | **Valley** | **Season** |
| --- | --- | --- | --- | --- | --- |
| 2011 | March | 1 | 1 | Nubri | Pre-monsoon |
| 2011 | April | 2 | 1 | Dudhkoshi | Pre-monsoon |
| 2011 | May | 3 | 1 | Olanchungola | Pre-monsoon |
| 2011 | July | 1 | 2 | Nubri | Monsoon |
| 2011 | August | 3 | 2 | Olanchungola | Monsoon |
| 2011 | September | 2 | 2 | Dudhkoshi | Post-monsoon |
| 2012 | April | 6 | 1 | Ghunsa | Pre-monsoon |
| 2012 | Apr/May | 3 | 3 | Olanchungola | Pre-monsoon |
| 2012 | June | 5 | 1 | Dudhkunda | Monsoon |
| 2012 | July | 2 | 3 | Dudhkoshi | Monsoon |
| 2012 | September | 4 | 1 | Tsum | Post-monsoon |
| 2012 | October | 1 | 3 | Nubri | Post-monsoon |
| 2012 | November/December | 6 | 2 | Ghunsa | Winter |
| 2013 | March | 4 | 2 | Tsum | Pre-monsoon |
| 2013 | April | 5 | 2 | Dudhkunda | Pre-monsoon |
