# Supplementary material for: Seasonal Changes in Bird Species and Feeding Guilds along Elevational Gradients of the Central Himalayas, Nepal
Source: PLoS One. 2016 Jul 1;11(7):e0158362. doi: 10.1371/journal.pone.0158362 (PMC4930183; doi:10.1371/journal.pone.0158362)
Supplement: S2 Table — The feeding guild and migration classification, based on descriptions available in Grimmett et al. (2000, 2011) and Basnet et al. (2016). Valleys 1 to 6 represent Nubri, Dudhkoshi, Olanchungola, Tsum, Dudhkunda, and Ghunsa, respectively. Feeding guilds are carnivores (C), herbivores (H), insectivores (I), nectarivores (N), and omnivores (O). Unexpected observations occurred for: Blue-capped Rock Thrush (Monticola cinclorhynchus), Large-billed Leaf Warbler (Phylloscopus magnirostris), White-browed Fulvetta (Alcippe vinipectus), Rufous Sibia (Heterphasia capistrata), Golden-spectacled Warbler (Seicercus burkii), and Spotted Nutcraker (Nucifraga caryocatactes), all of which were recorded at least 300 m above their upper elevation limits as described in Grimmett et al. (2000). The common lowland species Back-shouldered Kite (Elanus caeruleus) was recorded in DDV at 3000 m a.s.l., although Grimmett et al. (2000) indicate that the species is located up to 1550 m a.s.l. as a summer visitor in the Kathmandu valley. (DOC) [file pone.0158362.s006.doc]

**Supporting Information**

**S2 Table. List of overall 178 species recorded and list of unexpected observations in six valleys of the Central Himalayas, Nepal.** The feeding guild and migration classification, based on descriptions available in Grimmett *et al.* (2000, 2011) and Basnet *et al.* (2016). Valleys 1 to 6 represent Nubri, Dudhkoshi, Olanchungola, Tsum, Dudhkunda, and Ghunsa, respectively. Feeding guilds are carnivores (C), herbivores (H), insectivores (I), nectarivores (N), and omnivores (O). Unexpected observations occurred for: Blue-capped Rock Thrush (*Monticola cinclorhynchus*)*,* Large-billed Leaf Warbler (*Phylloscopus magnirostris),* White-browed Fulvetta (*Alcippe vinipectus*)*,* Rufous Sibia (*Heterphasia capistrata*), Golden-spectacled Warbler (*Seicercus burkii*), and Spotted Nutcraker (*Nucifraga caryocatactes*), all of which were recorded at least 300 m above their upper elevational limits as described in Grimmett *et al.* (2000). The common lowland species Back-shouldered Kite (*Elanus caeruleus*) was recorded in DDV at 3000 m a.s.l., although Grimmett *et al.* (2000) indicate that the species is located up to 1550 m a.s.l. as a summer visitor in the Kathmandu valley.

| No. | Common Name | Scientific Name | Species Code | Feeding Guild | Migration Type | Valleys |
| --- | --- | --- | --- | --- | --- | --- |
| 1 | Northern Goshawk | *Accipiter gentilis* | AccGen | C | Winter visitor | 5 |
| 2 | Eurasian Sparrowhawk | *Accipiter nisus* | AccNis | C | Resident and winter visitor | 2 |
| 3 | Besra | *Accipiter virgatus* | AccVir | C | Resident | 3 |
| 4 | Hoary-throated Barwing | *Actinodura nipalensis* | ActNip | I | Resident | 5 |
| 5 | Black-throated Tit | *Aegithalos concinnus* | AegCon | I | Resident | 1-6 |
| 6 | Rufous-fronted Tit | *Aegithalos iouschistos* | AegIou | I | Resident | 5 |
| 7 | Mrs Gould's Sunbird | *Aethopyga gouldiae* | AetGou | N | Resident | 1,4,5 |
| 8 | Fire-tailed Sunbird | *Aethopyga ignicauda* | AetIgn | N | Resident | 1-6 |
| 9 | Green-tailed Sunbird | *Aethopyga nipalensis* | AetNip | N | Resident | 1-6 |
| 10 | Rufous-winged Fulvetta | *Alcippe castaneceps* | AlcCas | I | Resident | 1,3-6 |
| 11 | White-browed Fulvetta | *Alcippe vinipectus* | AlcVin | I | Resident | 1,3-6 |
| 12 | Olive-backed Pipit | *Anthus hodgsoni* | AntHod | I | Resident | 1-6 |
| 13 | Rosy Pipit | *Anthus roseatus* | AntRos | I | Resident | 1-6 |
| 14 | Paddyfield Pipit | *Anthus rufulus* | AntRuf | I | Resident | 1,3-6 |
| 15 | Upland Pipit | *Anthus sylvanus* | AntSyl | I | Resident | 2 |
| 16 | Tree Pipit | *Anthus trivialis* | AntTri | I | Winter visitor and passage migrant | 2,5 |
| 17 | Golden Eagle | *Aquila chrysaetos* | AquChr | C | Resident | 1 |
| 18 | Common Buzzard | *Buteo buteo* | ButBut | C | Winter visitor and passage migrant | 6 |
| 19 | Long-legged Buzzard | *Buteo rufinus* | ButRuf | C | Winter visitor and passage migrant | 6 |
| 20 | Common Rosefinch | *Carpodacus erythrinus* | CarEry | H | Resident and winter visitor | 1-6 |
| 21 | Dark-breasted Rosefinch | *Carpodacus nipalensis* | CarNip | H | Resident | 5 |
| 22 | Beautiful Rosefinch | *Carpodacus pulcherrimus* | CarPul | H | Resident | 1-6 |
| 23 | Red-fronted Rosefinch | *Carpodacus puniceus* | CarPun | H | Resident | 3,5,6 |
| 24 | Spot-winged Rosefinch | *Carpodacus rodopeplus* | CarRo2 | H | Resident | 2,3 |
| 25 | Pink-browed Rosefinch | *Carpodacus rodochrous* | CarRod | H | Resident | 2-5 |
| 26 | Blanford's Rosefinch | *Carpodacus rubescens* | CarRub | H | Resident | 1 |
| 27 | Yellow-breasted Greenfinch | *Carduelis spinoides* | CarSpi | H | Resident | 1-5 |
| 28 | White-browed Rosefinch | *Carpodacus thura* | CarThu | H | Resident | 2,3,5,6 |
| 29 | Fire-capped Tit | *Cephalopyrus flammiceps* | CepFla | I | Resident and uncertain migrant | 1,2 |
| 30 | Eurasian Treecreeper | *Certhia familiaris* | CerFam | I | Resident | 1-6 |
| 31 | Rusty-flanked Treecreeper | *Certhia nipalensis* | CerNip | I | Resident | 3,6 |
| 32 | White-capped Water Redstart | *Chaimarrornis leucocephalus* | ChaLeu | I | Resident | 1-6 |
| 33 | Orange-bellied Leafbird | *Chloropsis hardwickii* | ChlHar | H | Resident | 3 |
| 34 | Brown Dipper | *Cinclus pallasii* | CinPal | C | Resident | 5,6 |
| 35 | Speckled Wood Pigeon | *Columba hodgsonii* | ColHod | H | Resident | 1,4,5 |
| 36 | Snow Pigeon | *Columba leuconota* | ColLeu | H | Resident | 1-6 |
| 37 | Rock Pigeon | *Columba livia* | ColLiv | H | Resident | 1-6 |
| 38 | Hill Pigeon | *Columba rupestris* | ColRup | H | Resident | 1 |
| 39 | Common Raven | *Corvus corax* | CorCor | O | Resident | 1 |
| 40 | Large-billed Crow | *Corvus macrorhynchos* | CorMac | O | Resident | 1-6 |
| 41 | Eurasian Cuckoo | *Cuculus canorus* | CucCan | I | Summer visitor | 1,5 |
| 42 | Lesser Cuckoo | *Cuculus poliocephalus* | CucPol | I | Summer visitor | 5 |
| 43 | Oriental Cuckoo | *Cuculus saturatus* | CucSat | I | Summer visitor | 3,5 |
| 44 | Grey-headed Canary Flycatcher | *Culicicapa ceylonensis* | CulCey | I | Resident and partial migrant | 1-6 |
| 45 | Darjeeling Woodpecker | *Dendrocopos darjellensis* | DenDar | I | Resident | 2,3,5 |
| 46 | Grey Treepie | *Dendrocitta formosae* | DenFor | H | Resident | 3,6 |
| 47 | Fire-breasted Flowerpecker | *Dicaeum ignipectus* | DicIgn | N | Resident | 1-6 |
| 48 | Ashy Drongo | *Dicrurus leucophaeus* | DicLeu | I | Resident and partial migrant | 2,3,5,6 |
| 49 | Black Drongo | *Dicrurus macrocercus* | DicMac | I | Resident | 3 |
| 50 | Yellow-bellied Flowerpecker | *Dicaeum melanoxanthum* | DicMel | N | Resident | 2,3 |
| 51 | Black-shouldered Kite | *Elanus caeruleus* | ElaCae | C | Resident and summer visitor | 5 |
| 52 | Little Forktail | *Enicurus scouleri* | EniSco | I | Resident | 5 |
| 53 | Verditer Flycatcher | *Eumyias thalassina* | EumTha | I | Resident and partial migrant | 1, 2, 3, 5, 6 |
| 54 | Common Kestral | *Falco tinnunculus* | FalTin | C | Resident, winter visitor and passage migrant | 1,3,4,6 |
| 55 | Rufous-gorgetted Flycatcher | *Ficedula strophiata* | FicStr | I | Resident | 3-6 |
| 56 | Ultramarine Flycatcher | *Ficedula superciliaris* | FicSup | I | Summer visitor | 4, 5 |
| 57 | Slaty-blue Flycatcher | *Ficedula tricolor* | FicTri | I | Resident | 1,3 |
| 58 | Black-faced Laughingthrush | *Garrulax affinis* | GarAff | O | Resident | 1-6 |
| 59 | White-throated Laughingthrush | *Garrulax albogularis* | GarAlb | O | Resident | 1,5 |
| 60 | Chestnut-crowned Laughingthrush | *Garrulax erythrocephalus* | GarEry | I | Resident | 2,4,5,6 |
| 61 | Streaked Laughingthrush | *Garrulax lineatus* | GarLin | O | Resident | 1-6 |
| 62 | Spotted Laughingthrush | *Garrulax ocellatus* | GarOce | O | Resident | 2,3,5,6 |
| 63 | Blue-winged Laughingthrush | *Garrulax squamatus* | GarSqu | O | Resident | 5 |
| 64 | Striated Laughingthrush | *Garrulax striatus* | GarStr | O | Resident | 3,5,6 |
| 65 | Scaly Laughingthrush | *Garrulax subunicolor* | GarSub | O | Resident | 2,5 |
| 66 | Variegated Laughingthrush | *Garrulax variegatus* | GarVar | O | Resident | 1,4 |
| 67 | Collared Owlet | *Glaucidium brodiei* | GlaBro | C | Resident | 4 |
| 68 | Bearded Vulture | *Gypaetus barbatus* | GypBar | C | Resident | 1,2 |
| 69 | Himalayan Griffon | *Gyps himalayensis* | GypHim | C | Resident | 1,4,6 |
| 70 | Scarlet Finch | *Haematospiza sipahi* | HaeSip | H | Resident | 2 |
| 71 | Rufous Sibia | *Heterphasia capistrata* | HetCap | O | Resident | 1-6 |
| 72 | Large Hawk Cuckoo | *Hierococcyx sparverioides* | HieSpa | I | Summer visitor | 3,5,6 |
| 73 | Barn Swallow | *Hirundo rustica* | HirRus | I | Resident and summer visitor | 4 |
| 74 | White-bellied Redstart | *Hodgsonius phaenicuroides* | HodPha | I | Summer and winter visitor | 1 |
| 75 | Black Bulbul | *Hypsipetes leucocephalus* | HypLeu | H | Resident | 1-6 |
| 76 | Mountain Bulbul | *Hypsipetes mcclellandii* | HypMcc | H | Resident | 1 |
| 77 | Black Eagle | *Ictinaetus malayensis* | IctMal | C | Resident | 6 |
| 78 | Blood Pheasant | *Ithaginis cruentus* | IthCru | H | Resident | 1-6 |
| 79 | Long-tailed Shrike | *Lanius schach* | LanSch | C | Resident | 1-6 |
| 80 | Grey-backed Shrike | *Lanius tephronotus* | LanTep | C | Resident | 1-6 |
| 81 | Plain Mountain Finch | *Leucosticte nemoricola* | LeuNem | H | Resident | 4,5 |
| 82 | Himalayan Monal | *Lophophorus impejanus* | LopImp | H | Resident | 4,6 |
| 83 | Kalij Pheasant | *Lophura leucomelanos* | LopLeu | H | Resident | 1-6 |
| 84 | Red Crossbill | *Loxia curvirostra* | LoxCur | H | Resident | 5,6 |
| 85 | Indian Blue Robin | *Luscinia brunnea* | LusBru | I | Summer visitor | 5 |
| 86 | Great Barbet | *Megalaima virens* | MegVir | H | Resident | 1 |
| 87 | Chestnut-tailed Minla | *Minla strigula* | MinStr | I | Resident | 3,5,6 |
| 88 | Blue-capped Rock Thrush | *Monticola cinclorhynchus* | MonCin | I | Summer visitor | 6 |
| 89 | Chestnut-bellied Rock Thrush | *Monticola rufiventris* | MonRuf | I | Resident | 1,3,5,6 |
| 90 | White Wagtail | *Motacilla alba* | MotAlb | I | Resident, winter and  passage migrant | 1,2,4, |
| 91 | Grey Wagtail | *Motacilla cinerea* | MotCin | I | Resident | 1-6 |
| 92 | Citrine Wagtail | *Motacilla citreola* | MotCit | I | Winter visitor and passage migrant | 3 |
| 93 | Yellow Wagtail | *Motacilla flava* | MotFla | I | Winter visitor | 3,5 |
| 94 | Rusty-tailed Flycatcher | *Muscicapa ruficauda* | MusRuf | I | Summer visitor | 3 |
| 95 | Dark-sided Flycatcher | *Muscicapa sibirica* | MusSib | I | Summer visitor | 1-6 |
| 96 | Collared Grosbeak | *Mycerobas affinis* | MycAff | H | Resident | 4,6 |
| 97 | White-winged Grosbeak | *Mycerobas carnipes* | MycCar | H | Resident | 1-6 |
| 98 | White-tailed Robin | *Myiomela leucura* | MyiLeu | I | Resident | 3 |
| 99 | Blue Whistling Thrush | *Myophonus caeruleus* | MyoCae | O | Resident | 1-6 |
| 100 | Small Niltava | *Niltava magrigoriae* | NilMag | I | Resident | 5 |
| 101 | Rufous-bellied Niltava | *Niltava sundara* | NilSun | I | Resident | 5 |
| 102 | Spotted Nutcraker | *Nucifraga caryocatactes* | NucCar | H | Resident | 1-6 |
| 103 | Desert Wheatear | *Oenanthe deserti* | OenDes | I | Summer visitor | 3 |
| 104 | Coal Tit | *Parus ater* | ParAte | I | Resident | 1-6 |
| 105 | Grey- crested Tit | *Parus dichrous* | ParDic | I | Resident | 1-6 |
| 106 | Great Tit | *Parus major* | ParMaj | I | Resident | 2 |
| 107 | Spot-winged Tit | *Parus melanolophus* | ParMel | I | Resident | 1 |
| 108 | Green-backed Tit | *Parus monticolus* | ParMon | I | Resident | 1-6 |
| 109 | Black-throated Parrotbill | *Paradoxornis nipalensis* | ParNip | I | Resident | 6 |
| 110 | Rufous-vented Tit | *Parus rubidiventris* | ParRub | I | Resident | 1-6 |
| 111 | Eurasian Tree Sparrow | *Passer montanus* | PasMon | H | Resident | 2,4 |
| 112 | Long-tailed Minivet | *Pericrocotus ethologus* | PerEth | I | Resident | 1-6 |
| 113 | Scarlet Minivet | *Pericrocotus flammeus* | PerFla | I | Resident | 6 |
| 114 | Blue-capped Redstart | *Phoenicurus coeruleocephalus* | PhoCoe | I | Resident | 4 |
| 115 | Blue-fronted Redstart | *Phoenicurus frontalis* | PhoFro | I | Resident | 1-6 |
| 116 | Hodgson’s Redstart | *Phoenicurus hodgsoni* | PhoHod | I | Winter visitor | 1,5 |
| 117 | Black Redstart | *Phoenicurus ochruros* | PhoOch | I | Resident | 1,2 |
| 118 | White-throated Redstart | *Phoenicurus schisticeps* | PhoSch | I | Resident and winter visitor | 4,6 |
| 119 | Tickell's Leaf Warbler | *Phylloscopus affinis* | PhyAff | I | Resident | 1,2,4,5 |
| 120 | Lemon-rumped Warbler | *Phylloscopus chloronotus* | PhyChl | I | Resident | 1-6 |
| 121 | Smoky Warbler | *Phylloscopus fuligiventer* | PhyFul | I | Resident | 1 |
| 122 | Hume's Warbler | *Phylloscopus humei* | PhyHum | I | Resident | 1-6 |
| 123 | Ashy-throated Warbler | *Phylloscopus maculipennis* | PhyMac | I | Resident | 1-6 |
| 124 | Large-billed Leaf Warbler | *Phylloscopus magnirostris* | PhyMag | I | Summer visitor | 3,5,6 |
| 125 | Western-crowned Warbler | *Phylloscopus occipitalis* | PhyOcc | I | Winter visitor | 1,2 |
| 126 | Buff-barred Warbler | *Phylloscopus pulcher* | PhyPul | I | Resident | 1,2,3,6 |
| 127 | Blyth's Leaf Warbler | *Phylloscopus reguloides* | PhyReg | I | Resident | 4 |
| 128 | Greenish Warbler | *Phylloscopus trochiloides* | PhyTro | I | Summer, winter and passage migrant | 1-6 |
| 129 | Scaly-bellied Woodpecker | *Picus squamatus* | PicSqu | I | Resident | 1,4,5 |
| 130 | Scaly-breasted Wren Babbler | *Pnoepyga albiventer* | PnoAlb | I | Resident | 3 |
| 131 | Nepal Wren Babbler | *Pnoepyga immaculata* | PnoImm | I | Resident | 5 |
| 132 | Striated Prinia | *Prinia criniger* | PriCri | I | Resident | 1,4,6 |
| 133 | Crimson-browed Rosefinch | *Propyrrhula subhimachala* | ProSub | H | Resident | 3 |
| 134 | Maroon-backed Accentor | *Prunella immaculata* | PruAtr | O | Winter visitor | 1 |
| 135 | Alpine Accenter | *Prunella collaris* | PruCol | O | Resident | 6 |
| 136 | Brown Accentor | *Prunella fulvescens* | PruFul | O | Resident | 5 |
| 137 | Altai Accentor | *Prunella himalayana* | PruHim | O | Winter visitor | 4 |
| 138 | Rufous-breasted Accentor | *Prunella strophiata* | PruStr | H | Resident | 1,6 |
| 139 | Green Shrike Babbler | *Pteruthius xanthochlorus* | PteXan | I | Resident | 4 |
| 140 | Himalayan Bulbul | *Pycnonotus leucogenys* | PycLeu | H | Resident | 1,3,4,6 |
| 141 | Red-headed Bullfinch | *Pyrrhula erythrocephala* | PyrEry | H | Resident | 1,2,3,4 |
| 142 | Yellow-billed Chough | *Pyrrhocorax graculus* | PyrGra | O | Resident | 2,4 |
| 143 | Brown Bullfinch | *Pyrrhula nipalensis* | PyrNip | H | Resident | 4 |
| 144 | Red-billed Chough | *Pyrrhocorax pyrrhocorax* | PyrPyr | O | Resident | 1,2,4,5 |
| 145 | Goldcrest | *Regulus regulus* | RegReg | I | Resident | 1 |
| 146 | Yellow-bellied Fantail | *Rhipidura hypoxantha* | RhiHyp | I | Resident | 1-6 |
| 147 | Plumbeous Water Redstart | *Rhyacornis fuliginosa* | RhyFul | I | Resident | 3,4,5,6 |
| 148 | Grey Bushchat | *Saxicola ferrea* | SaxFer | I | Resident | 1-6 |
| 149 | Golden Spectacled Warbler | *Seicercus burkii* | SeiBur | I | Resident | 5 |
| 150 | Chestnut-crowned Warbler | *Seicercus castaniceps* | SeiCas | I | Resident | 3 |
| 151 | Whistler's Warbler | *Seicercus whistleri* | SeiWhi | I | Resident | 1-6 |
| 152 | Grey-hooded Warbler | *Seicercus xanthoschistos* | SeiXan | I | Resident | 1-6 |
| 153 | White-tailed Nuthatch | *Sitta himalayensis* | SitHim | I | Resident | 1-6 |
| 154 | Crested Serpent Eagle | *Spilornis cheela* | SpiChe | C | Resident | 3 |
| 155 | Black-chinned Babbler | *Stachyris pyrrhops* | StaChr | I | Resident | 4 |
| 156 | Spotted Dove | *Streptopelia chinensis* | StrChi | H | Resident | 1 |
| 157 | Oriental Turtle Dove | *Streptopelia orientalis* | StrOri | H | Resident | 1-6 |
| 158 | Lesser Whitethroat | *Sylvia curruca* | SylCur | I | Winter visitor and passage migrant | 4 |
| 159 | Golden Bush Robin | *Tarsiger chrysaeus* | TarChr | I | Resident | 3,5 |
| 160 | Orange-flanked Bush Robin | *Tarsiger cyanurus* | TarCya | I | Resident | 1-6 |
| 161 | Rufous-breasted Bush Robin | *Tarsiger hyperythrus* | TarHyp | I | Resident | 4 |
| 162 | White-browed Bush Robin | *Tarsiger indicus* | TarInd | I | Resident | 5,6 |
| 163 | Satyr Tragopan | *Tragopan satyra* | TraSat | H | Resident | 1,3,5 |
| 164 | Wedge-tailed Green Pigeon | *Treron sphenura* | TreSph | H | Resident | 1,3 |
| 165 | Winter Wren | *Troglodytes troglodytes* | TroTro | I | Resident | 3-6 |
| 166 | White-collared Blackbird | *Turdus albocinctus* | TurAlb | O | Resident | 1,3,5,6 |
| 167 | Grey-winged Blackbird | *Turdus boulboul* | TurBou | O | Resident | 1,5 |
| 168 | Kessler's Thrush | *Turdus kessleri* | TurKes | O | Winter visitor | 3 |
| 169 | Eurasian Blackbird | *Turdus merula* | TurMer | O | Winter visitor and passage migrant | 1 |
| 170 | Dark-throated Thrush | *Turdus ruficollis* | TurRuf | O | Winter visitor | 1,2 |
| 171 | Common Hoopoe | *Upupa epops* | UpuEpo | I | Resident | 1, 2,3,4,6 |
| 172 | Yellow-billed Blue Magpie | *Urocissa flavirostris* | UroFla | O | Resident | 1-6 |
| 173 | Whiskered Yuhina | *Yuhina flavicollis* | YuhFla | I | Resident | 1-3 |
| 174 | Stripe-throated Yuhina | *Yuhina gularis* | YuhGul | I | Resident | 2,3,4,5 |
| 175 | Rufous-vented Yuhina | *Yuhina occipitalis* | YuhOcc | I | Resident | 2,3,5,6 |
| 176 | Long-tailed Thrush | *Zoothera dixoni* | ZooDix | I | Resident | 4 |
| 177 | Long-billed Thrush | *Zoothera monticola* | ZooMon | I | Resident | 3 |
| 178 | Oriental White-eye | *Zosterops palpebrosus* | ZosPal | I | Resident | 4 |
